# Progress in the Application of Nanoparticles and Graphene as Drug Carriers and on the Diagnosis of Brain Infections

**DOI:** 10.3390/molecules26010186

**Published:** 2021-01-02

**Authors:** Mahmood Barani, Mahwash Mukhtar, Abbas Rahdar, Ghasem Sargazi, Anna Thysiadou, George Z. Kyzas

**Affiliations:** 1 Department of Chemistry, Shahid Bahonar University of Kerman, Kerman 76169-14111, Iran; mahmoodbarani7@gmail.com; 2 Faculty of Pharmacy, Institute of Pharmaceutical Technology and Regulatory Affairs, University of Szeged, 6720 Szeged, Hungary; mukhtar.mahwash@pharm.u-szeged.hu; 3 Department of Physics, Faculty of Science, University of Zabol, Zabol 538-98615, Iran; 4 Noncommunicable Diseases Research Center, Bam University of Medical Science, Bam 5166-15731, Iran; g.sargazi@gmail.com; 5 Department of Chemistry, International Hellenic University, 65404 Kavala, Greece; thysiadou@hotmail.com

**Keywords:** nanomaterials, brain, meningitis, treatment, diagnosis

## Abstract

The blood–brain barrier (BBB) is the protective sheath around the brain that protects the sensitive microenvironments of the brain. However, certain pathogens, viruses, and bacteria disrupt the endothelial barrier and cause infection and hence inflammation in meninges. Macromolecular therapeutics are unable to cross the tight junctions, thereby limiting their bioavailability in the brain. Recently, nanotechnology has brought a revolution in the field of drug delivery in brain infections. The nanostructures have high targeting accuracy and specificity to the receptors in the case of active targeting, which have made them the ideal cargoes to permeate across the BBB. In addition, nanomaterials with biomimetic functions have been introduced to efficiently cross the BBB to be engulfed by the pathogens. This review focuses on the nanotechnology-based drug delivery approaches for exploration in brain infections, including meningitis. Viruses, bacteria, fungi, or, rarely, protozoa or parasites may be the cause of brain infections. Moreover, inflammation of the meninges, called meningitis, is presently diagnosed using laboratory and imaging tests. Despite attempts to improve diagnostic instruments for brain infections and meningitis, due to its complicated and multidimensional nature and lack of successful diagnosis, meningitis appears almost untreatable. Potential for overcoming the difficulties and limitations related to conventional diagnostics has been shown by nanoparticles (NPs). Nanomedicine now offers new methods and perspectives to improve our knowledge of meningitis and can potentially give meningitis patients new hope. Here, we review traditional diagnosis tools and key nanoparticles (Au-NPs, graphene, carbon nanotubes (CNTs), QDs, etc.) for early diagnosis of brain infections and meningitis.

## 1. Introduction

Prostate viruses, bacteria, fungi, protozoa, and parasites are susceptible to infections of the brain. The common infectious agents of acute meningitis are viruses (mainly enteroviruses HIV, herpes simplex viruses (HSV), and mumps virus) and bacteria (*Streprococcus pneumoniae*, *Neisseira. meningitidis*, *Streprococcus agalactiae*, *Haemophilus influenzae*, and *Listeria monocytogenes*) [1]. Less common causes of acute meningitis include parasites (*N. fowleri*, *S. stercoralis*, and *A. cantonensis*). Abnormal proteins, also called spongiform encephalopathy, known to be prions, are responsible for brain disorders. Other parts of the central nervous system, including the spinal cord, are often involved in brain infections. The brain and the spinal cord are usually protected from infection but often are seriously affected when infected. An inflammation of tissue layers in the brain and in the spinal cord can also cause infection. Usually, viral meningitis is less clinically severe than bacterial meningitis. The main cause of encephalitis is meningitis, caused by encephalitis and viruses [2]. Technically, meningoencephalitis refers to when both the brain and meninges are infected. The infection is usually not confined to one area in encephalitis and meningitis. It may happen throughout the brain and throughout the whole spinal cord, but in certain types of disorders, the infection is localized between the tissues that cover the brain—that is, a pocket of pus called empyemas (present in lungs and brain)—and the infection resembles boils, referred to as abscesses (anywhere in the body including the brain). *Aspergilla* (Fungi), *Toxoplasma gondi* (protozoa), and *Taenia solium* (parasites) may also produce a tumor in the brain, and their infections contain a cluster of organisms enclosed in a protective wall, and sometimes these infections trigger a misguided immune reaction to produce another type of disorder due to this autoimmune reaction results in inflammation. This disorder is called postinfectious encephalitis.

Meningitis can cause necrosis, decreased blood and cerebrospinal fluid (CSF) flow, and impaired central nervous system (CNS) functions. Death occurs from shock and other serious complications within hours of the appearance of symptoms like headache, fever, stiff neck, and altered mental status. Infectious meningitis is a serious CNS disease involving microbial inflammation of the meninges and occurs in every age group. Viruses and bacteria, as well as, rarely, fungi or parasites, cause it more frequently.

Even after antibiotic and anti-inflammatory therapy, mortality and morbidity of bacterial meningitis are very high. In recent decades, bacterial meningitis therapy has therefore been designed not only to kill the invading bacteria but to decompose the consequent inflammatory response. The combination of bacteriolytic E-lactam antibiotic antibiotics with corticosteroids is a current treatment of choice in non-infected adult patients with bacterial meningitis, according to the guidelines of the European Federation of Neurological Societies (EFNS) [3]. However, in terms of applicability and effectiveness, this treatment scheme has several drawbacks. New treatment strategies must therefore be developed for bacterial meningitis. Drugs such as Acyclovir (10 mg·kg^−1^ every 8 h), used for the treatment of Herpes simplex virus (HSV) and Varicella-Zoster virus (VZV); Ganciclovir (10 mg·kg^−1^ every 8 h) for Cytomagalovirus (CMV); and Ganciclovir (5 mg·kg^−1^ every 12 h) and Ampicillin (50 mg·kg^−1^ every 8 h) for Cytomegalovirus (CMV) are used for *E. coli*, and *L. monocytogenes*; Cefriaxone (2 every 12 h) for *S. pneumoniae*, *N. meningiditis*, and *H. influenza*; Vacomycin (5 mg·kg^−1^ every 12 h), Ceftazidime (5 g every 8 h) and Cefepime (2 g every 8 h) for *S. aureus* [4]. The current diagnostic procedure for meningitis is lumbar puncture (LP) or spinal tap, in which a small amount of CSF for testing is collected for virus and bacterial infection. There can be some complications, such as post-LP headache, infection, bleeding, cerebral herniation, back pain, late onset of epidermoid tumors of the thecal sac, and minor neurologic symptoms such as radicular pain [5]. It is recommended that computational tomographical (CT) of the head is obtained before LP given the potential of brain herniation in patients who are at risk of intracranial mass or intermediate shift [6]. The other medical tools such as MRI, CT scan, and Electroencephalogram (EEG) are also very useful for the treatment of meningitis. A wide range of antibiotics should be initiated and tapered as possible for treatment. Suspected encephalitis cases with antivirals should be considered empirically. Steroids have also been shown to improve neurological sequelae and meningitis hearing loss; however, in the case of viral encephalitis, these steroids are not recommended. Opportunistic CNS infections that need to be addressed in patients with considerable immunocompromises are of particular importance. Blocking Antibody Adjuvant Therapy offers a promising option in future patients with bacterial meningitis acquired throughout the community. The benefit of adjuvant corticosteroid therapy shows that the combination of antibiotics with pathophysiologically relevant drugs is key to improving clinical results. Dexamethasone was, however, only shown to have limited advantage. A translational approach that focuses on therapeutics that continues to be effective during the administration of antibiotics or even later on must be examined with priority to effectively change the patient outcome. In this regard, the use of nonbacteriolytic antibiotics and adjuvant inhibitors of matrix metalloproteinase appears to be promising.

The advance of nanotechnology has shown both nanosensor diagnosis and nanomaterial treatment to be efficient strategies for the healing of brain infections and meningitis [7]. The simple synthetic method, easy functionalization, capability of crossing cell membranes, increased drug release at the site of infection due to higher surface-to-volume ratio, ability to kill the pathogens by inhibiting multiple bacteriological targets, and, most importantly, the biological safety are some characteristic features of the nanoparticles.

With this in mind, in continuation of efforts from our group related to the synthesis of nanomaterials and investigation of their potential bioapplications, in the current work, we reviewed different nanomaterials applied to diagnosis and treatment of brain infections and meningitis.

## 2. Nanomaterials: Applications in Meningitis and Bacterial Infection of Brain

**Figure 1:**
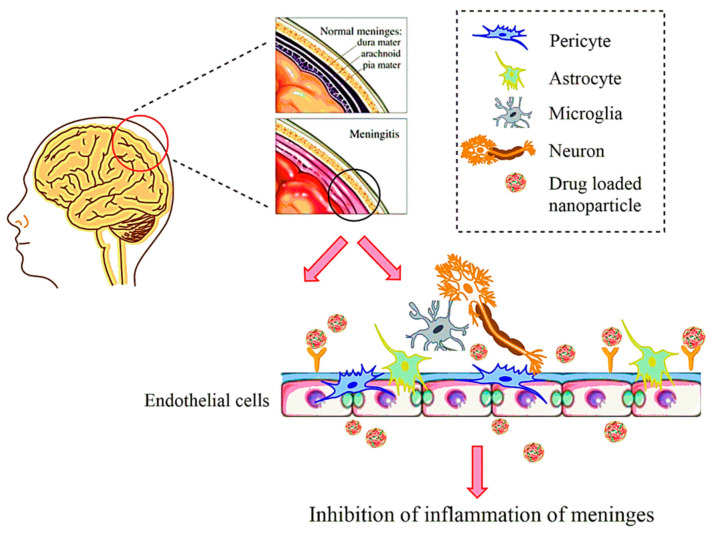
Schematic picture of nanoparticles acting on the endothelial cells of the brain for drug delivery in meningitis.

The advent of nanotechnology has created many opportunities in enhancing drug delivery across the BBB. The challenges in the permeation across the complex physiology of BBB in the pathological conditions can be met by exploiting the nanodimensional vehicles with large surface area [8]. Their enhanced stability after physical and chemical modifications creates endless possibilities for administering drugs across the BBB for inflammatory disorders. Numerous nanotechnology-based drug delivery vehicles are presently being used. The nanocarriers show improved bioavailability, are highly soluble and non-toxic, have long circulation life, are biodegradable, and allow sustained drug release [9]. Nanopharmaceuticals can lead to passive diffusion through transcellular route and receptor-mediated transport across the endothelial cells of the brain or the choroid plexus. However, the hydrophobic nanostructures may pass through the active diffusion across BBB [10]. Figure 1 shows the simple representation of drug delivery by nanocarriers in meningitis. A few nanocarriers used in the treatment of meningitis and bacterial infections are listed.

### 2.1. Lipid-Based Nanocarriers

Liposomes are nanostructures with membrane-penetrating properties. There are many methods for the preparation of liposomes to yield high entrapment efficiency and correct size. Because of the unique composition of liposomes, hydrophilic and hydrophobic drugs can be loaded into them [11]. Phosphoethanolamine-based lipid nanocarriers support the penetration of the encapsulated drug across the outer membrane of the Gram-negative bacteria to perform bactericidal action in meningitis [12]. Furthermore, liposomes are non-toxic because of phospholipids composition and therefore biodegradable and non-immunogenic. The intranasal route of administration was explored recently to deliver a levofloxacin and doxycycline combination to the brain for the treatment of meningitis. Solid lipid NPs were developed with a hot emulsification method. There was a significant increase in the concentration of drugs in the brain as compared to the free drug because of the BBB permeation properties of lipids, making it a promising therapy for meningitis [13]. Nanostructured lipid carriers loaded with ketoconazole were assessed for meningoencephalitis. Fluorescent dye was loaded in the nanostructures later to confirm the efficient uptake of nanocarriers in the *Cryptococcus neorformans*, the yeast responsible for meningoencephalitis. The lipid carriers took the olfactory pathway to reach the brain, as demonstrated by animal studies. There was a significant increase in the delivery of ketoconazole across the BBB, which contributed to the treatment [14]. The pharmacodynamics of the amphotericin B-loaded liposomes were investigated in murine and rabbit models of cryptococcal meningitis. A dose-dependent decline was observed in the fungal burden in the brain as displayed by immunohistochemistry studies, and elimination half-life was found to be 133 h. Moreover, the drug was found to be present in the leptomeninges, intra- and perivascular spaces [15]. Coccidioidal meningitis can also be treated by a similar liposomal formulation encapsulating amphotericin B and demonstrated lower nephrotoxicity and higher concentrations in the reticuloendothelial system as compared to free amphotericin B. The clinical study in the hospital setting proved a potential treatment option in patients with chronic meningitis and undertaking triazole therapy [16]. Similarly, another clinical study reported that administration of low-dose amphotericin B liposomes in combination with voriconazole was effective in *C. neoformans* meningitis. There was a significant increase in the BBB penetration of voriconazole. The drug was chosen because of its fungal cell membrane ergosterol disruption [17]. Currently, alternative novel fungal CYP inhibitors are being explored to improve the specificity to the target and to reduce toxicity. VT-1598 is one such novel fungal CYP51 inhibitor that uses tetrazole as the haem-iron binding moiety. Amphotericin B loaded liposomes were administered orally in combination with VT-1598 in the murine model of cryptococcal meningitis. The combination therapy was effective against *C. neoforms* and *C. gattii* [18].

**Figure 2:**
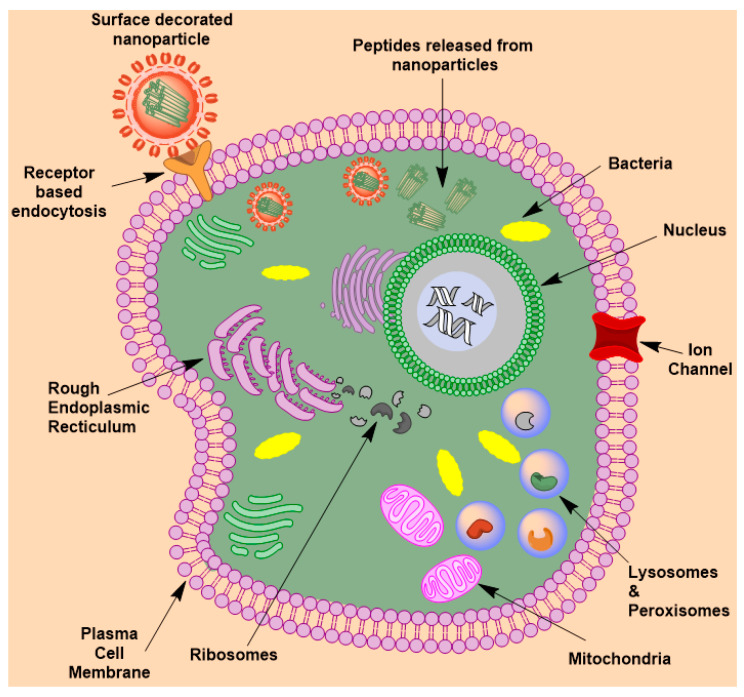
Receptor-mediated endocytosis of peptide encapsulated nanostructure across the cellular membrane.

Recent innovations have been introduced in the liposomal drug delivery for meningitis. One such approach is the use of cell-penetrating peptides attached to the surface of liposomes. Figure 2 shows the general mechanism of peptide release from the nanostructures by active targeted delivery to the cellular membrane. Tat (YGRKKRRQRRR) is a peptide sequence that improves the permeation of the liposomes across the cellular membrane [19]. Tat-decorated fusogenic liposomes were reported to have a bactericidal function. Fusogenic liposomes destabilize the bacteria based on the pH fluctuations. A study reported the Tat-functionalized fusogenic liposomes synthesized by the thin-lipid-film rehydration technique. Tat-functionalized liposomes exhibited bactericidal activity efficiently compared to non-functionalized liposomes. Tat-decorated liposomes inhibited up to 95% *S. pneumoniae* bacteria compared to non-functionalized liposomes. Cytotoxicity assay with astrocytes and endothelial cells demonstrated the safety profile of Tat-functionalized liposomes [20]. Lipid soluble compounds can penetrate the BBB by passive transcellular diffusion and hence can be the optimal drug delivery vehicle in the treatment of brain infections.

### 2.2. Metal Nanoparticles

Metal NPs are the nanoscaled vehicles used in the biomedical sciences. Gold and silver NPs are the few metallic NPs used in drug delivery. Gold nanoparticles (AuNPs) are the sub-colloidal metallic structures. AuNPs are diverse nanocarriers of smaller size with vast surface modification options. AuNPs have tunable physical and chemical properties [21]. The most promising applications of AuNPs are for antimicrobial and antipathogenic activities. The surface of gold nanoparticles can also be exploited for the adherence of proteins by the electrostatic interaction between the positively charged group of proteins and negatively charged citrate on the AuNPs surface. Currently, AuNPs are being extensively studied in the photodynamic therapy of brain diseases [22]. Brain-eating amoeba, *N. fowleri*, is the cause of lethal amoebic meningoencephalitis. Nanotechnology-based metallic structures have proved to be promising in brain infections. A recent study highlighted the effectiveness of cinnamic acid-loaded AuNPs. Nanocarriers were synthesized by reduction of tetrachloroauric acid and enhanced the penetration across BBB. Amoebicidal and cytopathogenicity assays demonstrated anti-amoebic properties. Cinnamic acid exhibited low toxicity because of its plant origin compared to other biologically derived compounds [23]. Another current study showed that the AuNPs prevented HSV-1 infection by crossing BBB. NPs were synthesized according to the generation type such as AuNPs with two sulfonate end groups, four sulfonate end groups, and eight sulfonate end groups. AuNPs exhibited HSV-1 infection through cellular or viral receptors. NPs also reverted the increased β-secretase activity. The histological studies in mice brains showed no signs of edema or necrosis. Hence the AuNPs reduced herpes-associated amyloid β-secretion [24].

In Europe, magnetic hyperthermia has been approved clinically for the treatment of brain tumors. However, the therapy still needs to be investigated for clinical effectiveness in brain infections [25]. For instance, metallic nanorods are being investigated presently for their potential to slow down the progression of brain pathologies like tumors by the induction of optical hyperthermia. The gold nanorods decorated with CD133 antibody, when irradiated with laser beam, resulted in the regression of tumor. Gold nanorods along with a continuous laser wave of 808 nm proved an optimistic therapy of glioblastoma multiforme by the induction of hyperthermia [26].

Similarly, silver nanoparticles (AgNPs) are also excellent drug delivery carriers. They have spherical structures with nanoscale dimensions and characteristic optical, electrical, and biological properties. AgNPs yield high stability, narrow size distribution, and solubility along with biocompatibility [27]. AgNPs have also been explored in amoebic meningoencephalitis. In one study, AgNPs were encapsulated with fluconazole, amphotericin B, and nystatin. Their size was reported to be around 100 nm. These NPs improved the anti-amoebic activity of the drugs compared to free drugs [28]. Presently, the potential of guanabenz has been studied because of its antipathogenic activity and ability to cross BBB. Conjugated AgNPs and AuNPs were constructed by one-phase reduction method to inhibit the brain-eating amoeba. AuNPs were found to be of narrow size distribution and smaller size as compared to AgNPs. The conjugated guanabenz NPs exhibited anti-amoebic activity against *N. fowleri* and *A. castellanii*. The NPs were found to be safe and completely compatible against human cells [29].

Recently, Anwar et al. demonstrated the use of green AuNPs and AgNPs for the treatment of brain infections. The novel AuNPs and AgNPs were loaded with hesperidin and naringin flavonoids as a treatment for brain-eating amoeba. The NPs were found to have antibacterial activity against neuropathogenic *E.coli* and methicillin-resistant *Staphylococcus aureus* (MRSA). Furthermore the AuNPs and AgNPs were amoebicidal against *N. fowleri* and *A. castellanii*. These green NPs exhibited minimal cytotoxicity and enhanced efficacy by crossing BBB [30]. Multiple bacteriological targets can be inhibited by the use of metallic NPs. However, the simplest method of synthesis and functionalization needs to be exploited to improve clinical efficacy. Different nanoparticles for the treatment of brain infections and meningitis are summarized in Table 1.

### 2.3. Polymeric Nanovehicles

Presently, polymeric nanoparticles are widely explored drug delivery vehicles in all infectious diseases because of their natural compatibility with the body cells and biodegradation. There has been an advancement in the polymeric role recently, and the concept of antimicrobial polymers has originated [43]. They mimic the chemical properties of the antimicrobial peptides that are native to the human body and defend against bacterial invasion [44].

**Figure 3:**
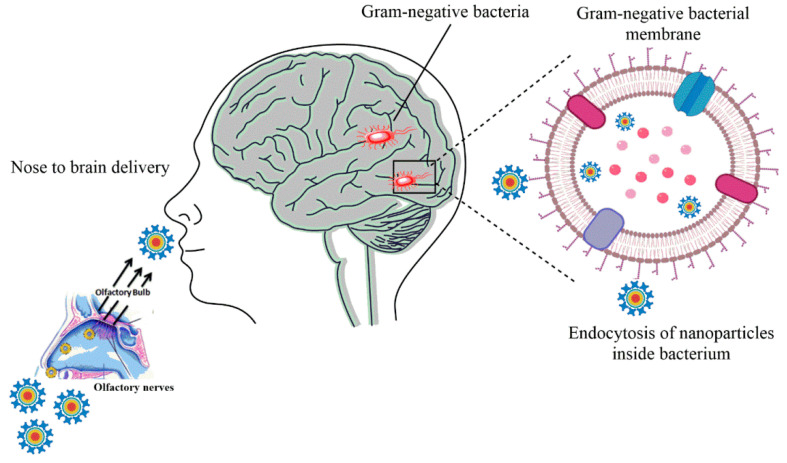
Nose-to-brain nanoparticle-mediated drug delivery for Gram-negative bacterial infection followed by endocytosis of nanoparticles in the bacterial cell.

A study explained the antifungal potential of polymeric nanoparticles with improved permeation across BBB. The transferrin receptor (TfR/CD71) is located on the BBB and can assist the endocytosis of the nanoparticles decorated with anti-transferrin antibody. Hence, the NPs were grafted with anti-TfR antibody (OX26) to enhance the receptor-mediated endocytosis of NPs in the endothelial cells of the brain. Amphotericin-B was loaded in the NPs of polymers (poly(lactic acid)) acid and PEG. Amphotericin-B-loaded NPs reduced the fungal infection and increased the lifespan of the mouse suffering from intracranial brain infection [45]. Presently, the nose-to-brain route is proving promising in the efficient delivery across BBB to treat brain infections like meningitis. Figure 3 shows the nose-to-brain route for the delivery of nanoparticles for the Gram-negative bacterial infections, as a general scheme. The polymeric nanoemulsions based on chitosan were exploited for the effective intranasal delivery of essential oils. The essential oils that were encapsulated in the nanoemulsions were Thymus vulgaris and Syzygium aromaticum. The polymeric nanoemulsions were effective against multi-drug-resistant Gram-negative microorganisms causing meningitis. The nanoemulsions were completely biocompatible because of the natural origin of the components of nanoemulsions [46]. TAT-modified cationic peptide PA-28 is gaining interest because of its antibacterial properties. PA-28 peptide was self-assembled into NPs, and the developed NPs exhibited the antibacterial activity against Gram-positive, Gram-negative, and drug-resistant bacteria. There was a remarked reduction in the bacterial strain across BBB. The bacterial growth was inhibited in the infected brain of the rats by the nanocarriers that also showed low hemolysis [47]. Another novel nanocarrier made of a con-covalent complex of methoxy poly (ethylene glycol)-poly(lactide)-poly(β-amino ester) (MPEG-PLA-PAE) was designed and loaded with amphotericin-B. The nanocomplex polymeric structures displayed high stability and activity against *C. neoformans* infection by the potential reduction of toxicity in the non-targeted organs [48]. Microemulsions encapsulating curcumin (CUR) and albendazole sulfoxide (ABZ-SO) were developed for intranasal administration in brain infection. The polymeric docosahexaenoic acid microemulsions enhanced the concentration of CUR and ABZ-SO across BBB. There was a significant increase of 10 folds in the permeation of the drug with the drug delivery vehicle [49]. An interesting hybrid nanostructure with polymeric and lipid composition was developed for the delivery of itraconazole across BBB for meningitis. The hydrophobic nature of the drug limits its use in brain diseases. Hence, bovine serum albumin NPs were synthesized, and the drug was loaded in their core followed by subsequent modification with borneol and PEG. The novel nanostructures significantly improved the cellular uptake of drugs in the *C. neoformans* across the BBB. Additionally, there was a two-fold increase in the distribution of the drug in the brain tissues [50].

### 2.4. Extracellular Membrane Vesicles (EMVs)

Membrane vesicles are widely shed by bacteria and pathogens. They are lipid bilayered nanostructures and are spherical in shape. Recently, they are being utilized to deliver cargoes to the site of action in the same bacteria, pathogen, or mammalian cells [51]. To elicit an immune response, trivalent native outer membrane vesicles (nOMVs) were used in bacterial meningitis. These nOMVs were derived from genetically modified *Neisseria meningitis* serogroup B. These smaller-scale nOMVs were evaluated for immunization in infant rhesus macaque models (IRMs). First, IRMs were immunized with nOMVs, and the antibody response was then observed for exogenous human serum complement-dependent SBA, which was promising [52]. In another similar study, nOMVs were prepared from meningococcal strains with genetically modified endotoxin and overexpressed Factor H binding protein (FHbp). The human complement-mediated serum bactericidal antibody response was measured in the mice immunized with nOMVs-FHbp as a vaccine against gonococcal and meningococcal strains. The response was three folds higher immunization than control FHbp vaccine [53]. It was found that after Listeria monocytogenes infection in the culture system, the microglia shed the extracellular traps (ETs), which comprise metallopeptidases (MMP9 and MMP12) and histones. These microglial ETs were found in the brain and were protected against *Listeria* meningitis. Interferon-γ could also stimulate the microglial ETs in the in vitro examination. Hence, this novel discovery has paved way for the deep exploration of the innate immune responses in humans for chronic illnesses [54].

### 2.5. Nanomicelles

Conventional antibiotic therapy has several limitations, including inefficient drug transport across the BBB. Hence, strategies such as micellar nanostructures should be exploited for meningitis and brain infections. Self-assembled nanosystems are called micelles and can incorporate drugs of different nature [55]. The nanomicelles possess structural, physical, chemical, and biological properties that make them suitable for the delivery of drugs across the BBB. The nanomicelles were engineered by different methods, but the recent methods include stimuli-responsive micelles that release the drug only in the presence of the stimulus such as light, magnetic field, acidic pH, and temperature [56].

PEGylated nanomicelles of bacitracin-A were synthesized and tested for antibacterial activity in pneumococcal meningitis. To further enhance the targeting, brain-targeting peptide (RVG_29_) and p-glycoprotein inhibitor (Pluronic^®^ P_85_ unimers) were conjugated to form a mixed micellar system. The mixed micellar system displayed high BBB permeation efficiency along with increased uptake in brain capillary endothelial cells. Optical imaging showed that micelles accumulated in the parenchymal cells of the brain via receptor-mediated transcytosis. The results from the animal studies were promising in the treatment of pneumococcal meningitis [57]. In another study, nanoscaled micelles were developed to penetrate the endothelial cells of BBB. Dehydroascorbic acid (DHA) was used to synthesize nanomicelles. The micelles were constructed by disulfide linkage to form the sheath around them to prevent leakage of encapsulated itraconazole. The drug was later released by high intracellular levels of glutathione once micelles were inside the cell. DHA-based micelles improved the concentration of itraconazole across BBB for effectiveness in intracranial infections [58]. A breakthrough research study focused on the vaccine Trumenba (bivalent rLP2086), which is licensed for meningococcal meningitis prevention in the USA. The vaccine demonstrated bactericidal properties by induction of antibodies against *Neisseria meningitidis* serogroup B. The vaccine chemistry reveals self-assembled lipoproteins forming micelles and polypeptide content. Moreover, the vaccine was found to be an agonist of toll-like receptor-2 because of the two O-liked fatty acids and the N-terminal lipid content of the micellar vaccine played role in the induction of immune response [59].

### 2.6. Other Nanosystems

Dendrimers are globule-like structures with nanodimensions. They may be divergent or convergent depending on the method of synthesis. The most commonly explored dendrimers are synthesized of poly (amidoamine) (PAMAM) and polypropylenimine (PPI). Peptide-based dendrimers mimic the antimicrobial peptides, cationic dendrimers target bacterial membranes, and glycodendrimers interfere with the adhesion to eukaryotic cell [60]. Pneumococcal infections can be treated by dendrimers by targeting choline-binding proteins (CBPs). Choline dendrimers were constructed with an affinity for the CBPs on the microglia. Once the pneumococcal cultures were incubated with dendrimers, they increased bacterial uptake by microglial cultures. The extent of phagocytosis was dependent on the dendrimers dose. The terminal choline-containing multivalent dendrimers were found promising in pneumococcal diseases [61]. Similarly, quantum dots (QDs) are uniquely nanostructured with peculiar optical and physical properties and are used in biomedicine, biosensing, and optronics. QDs possess high stability, solubility, and biocompatibility and offer multiple options for surface modifications [62]. Carbon QDs were found to be effective in the delivery of curcumin in meningitis and brain infections caused by enterovirus 71 (EV71). The results demonstrated the improved curcumin activity by the significant inhibition of viral activity. The intraperitoneal administration of the carbon QDs inhibited virus in the mice model [63].

## 3. Diagnosis of Brain Infections and Meningitis by Nanotechnology

**Figure 4:**
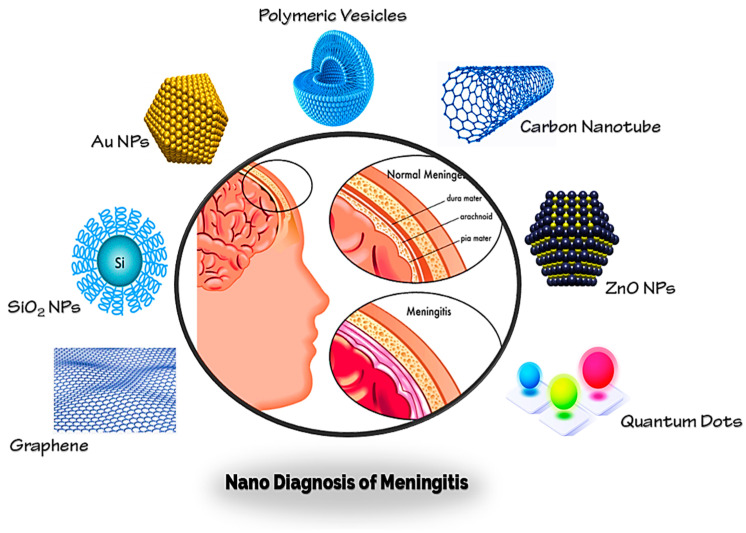
Different nanoparticles for detection of brain infection and meningitis.

Brain infections are relatively uncommon, but they are potentially dangerous and have a high mortality rate. Meningitis, known collectively as meninges, is an inflammatory condition of the protective membranes that surround the spinal cord and brain [64,65,66]. The most common signs are headache, fever, and discomfort of the neck [67]. Infection may be caused by bacteria, viruses, or other pathogens and, less often, by some medicines [68]. A lumbar puncture, where a syringe for a sample of cerebrospinal fluid (CSF) is injected into the spinal canal, may exclude and diagnose meningitis [69]. If there is a lump in the brain (abscess or tumor) or if the intracranial pressure (ICP) is increased, lumbar puncture is considered unsafe, as it may contribute to brain-herniated disc. A CT or MRI scan is advised before the lumbar puncture if anyone is at risk for either a mass or elevated ICP [70]. Blood check indicators for infections such as C-reactive protein, full blood count, and blood cultures are conducted if anyone is suspected of having meningitis [71,72]. Almost all of these diagnostic tools have struggled in clinical application, despite remarkable success in preclinical trials and conventional diagnosis, which may be attributed to the complicated nature of brain infections and meningitis [73]. There is therefore an essential need for the relevance of conventional methods to be re-examined and for novel therapeutic tactics to be created. The latest nanotechnology advances have created an unparalleled potential to enhance the diagnosis and early identification of meningitis and other brain diseases [8,74,75]. NPs are widely accepted as promising materials for diagnosis of meningitis brain infections because of intrinsically useful features such as elevated surface-to-volume ratio, simplicity of surface modification with preferred ligands, and capacity to cross biological barriers (such as the BBB) [57,76]. Some main nanoparticles for early diagnosis of brain infections and meningitis (Au NPs, Graphene, CNTs, QDs, etc.) are discussed in this analysis (Figure 4).

### 3.1. Graphene Oxide

Graphene oxide-based nanomaterials have excellent physicochemical characteristics and are now commonly used in various applications for medicinal diagnosis. In addition, the use of graphene oxide has significantly enhanced the sensitivity and accuracy of new nanosensors [77,78].

Bacterial meningitis is a worldwide infectious disease with high mortality and morbidity (30-60% in developed countries) and becomes lethal within 24 h if not adequately treated [79]. The two major pathogens causing bacterial meningitis are *Streptococcus pneumoniae* (*S. pneumoniae*) and *Neisseria meningitidis* (*N. meningitidis*) [80]. In this context, Dou et al. generated a microfluidic system based on poly (methyl methacrylate)/paper composite CD-like for multimodal quantitative LAMP monitoring (m-mqLAMP). The nanoplatform had both ssDNA probe-functionalized graphene oxide nanosensors and LAMP amplification in one integrated system. [81]. A simple superior approach was offered by the incorporated nanosensors and mLAMP reactions on the device to resolve the challenges of traditional mLAMP in defining and quantifying different targets. Without depending on any pneumatic values and peripheral pumps, the rotary design of a CD-shaped nanoplatform allowed a quick and smooth communication between the mLAMP module and the biosensor diagnosis module. Two mentioned bacterial pathogens were successfully detected using m-mqLAMP, with high precision, illustrating the prototype success. The LODs for Meningitidis and Pneumoniae was obtained with 12 copies and 6 copies per experiment, respectively.

Tuberculosis (TB) is an infectious and serious disease that needs a highly responsive and precise diagnostic device. Mycobacterium tuberculosis causes tuberculous meningitis [82]. Perumal et al. grown nano-rods of gold (Au) by chemical vapor deposition on 3D graphene used it for diagnosis of *Mycobacterium tuberculosis* [83]. The unpredictably distributed and streaked 3D graphene structure offered a route to self-assemble Au NPS and shape rod-like configurations on the 3D graphene surface. The impedimetric sensing of DNA sequence of TB by Au NPS/3D graphene nanocomposite showed a surprisingly large linear detection range (10 fM to 0.1 μM), demonstrating the capacity to identify concentration of femtomolar for targeted DNA.

### 3.2. SiO_2_-NPs

**Figure 5:**
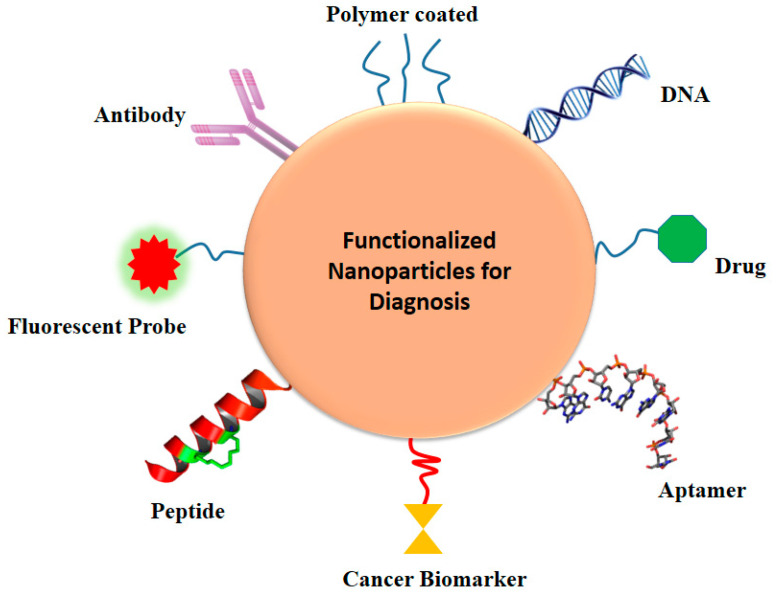
Schematic representation of multifunctional NPs in the diagnosis of brain infections.

Nanomaterials-based silica have been commonly used in diseases and disorders for diagnostic and therapeutic goals. The unique surface of silica NPs enables the conjugation of multiple biomolecules such as nucleic acids and proteins [84]. The invention of the CRISPR-Cas9 RNA-guided DNA nuclease has allowed the selective modification of sequences from various species, but the continuous nature of genome alteration also raises a barrier. Phaneuf et al. reported the ability of fluorescence-based multifunctional silica NPs (1000 nm) (attached to antibody) for diagnosis of Cas9 from *N. meningitidis*, *S. aureus*, *S. pyogenes,* and *S. thermophiles* (Figure 5) [85]. A responsive and simultaneous assessment of both the Cas9 protein and the activity in a single biological sample was seen in this study. They also noted that the phage-derived anti-CRISPR protein AcrIIC1, which attaches many species to Cas9, could be utilized as a catch reagent to increase the number of infection species diagnoses.

### 3.3. Gold Nanoparticles

In medical diagnostics, Au NPs are commonly employed since they have many useful effects: they are inert, their size and shape can be effectively controlled by differing reaction procedures, their surfaces can be easily functionalized, stable colloids can easily be formed, and they have low cytotoxicity [86,87].

Efforts were made to produce fast, cheap, and simole DNA testing, particularly in the field of brain disease diagnostics. Najian et al. mentioned the production of label-based Au NPs lateral flow dipstick nanosensor multiplex loop mediated isothermal amplification (m-LAMP) for the diagnosis of pathogenic Leptospira [88]. Leptospirosis is an infection caused by Leptospira bacteria in the blood. Common symptoms can vary from mild (headaches, muscle pain, and fevers) to extreme (lung bleeding or meningitis) [89]. Extra incubation steps and probe-hybridization were omitted in their suggested nanosensor. When evaluated with 13 typical pathogenic Leptospira species, 2 intermediate Leptospira species, 1 non-pathogenic Leptospira species, and 28 other bacteria species, the accuracy of this biosensor experiment was 100 percent. This analysis revealed that, at very low concentrations, this nanosensor can detect DNA.

In a similar study, Jiang et al. developed a facile, quick and responsive visual and POC detection approach for *Cronobacter* spp., *Salmonella* spp., and *S.* aureus in powdered infant formula (PIF) based on lateral flow dipstick (LFD) integrated with mLAMP [90]. Serious blood infections or meningitis and other inflammations of the brain and spine can result from Cronobacter bacteria [91]. LFD was found in a large number of altered amplicons. In this experiment, one end of the amplicon was bound to the anti-FITC Ab on the surface of Au NPs and the other end to streptavidin. The device’s visual inspection was focused on the existence of a red band created by the aggregation of composite sandwiches. The LODs for *S. aurous*, *Cronobacter* spp. and *Salmonella* spp. in this LFD-mLAMP test was 3.4, 2.6 and 4.2 cfu/g, respectively. In less than 1 h, the entire process was completed.

In another study, Patel et al. described an electrochemical DNA nanosensor on a glass electrode covered by Au NPs and immobilizing with oligonucleotide (ssDNA) probe for meningitis diagnosis [92]. The Au/DNA electrode detected complementary DNA in the range of 7–42 ng/μL in just 5 min with a response time of 60 s, and when stored at 4 °C, the electrode is stable for around 4 months. With a regression coefficient (R) of 0.917, the responsiveness of the Au/dsDNA electrode reached 115.8 μA/ng.

### 3.4. ZnO Nanoparticles

Recently, zinc oxide nanoparticles have become especially interesting in the field of diagnosis. Zinc oxide also has some promising potentials such as improved biocompatibility, antifungal, antiviral, antibacterial, and anti-cancer characteristics [93,94].

Tak et al. developed a multi-walled carbon nanotube (CNT) and ZnO NPs nanocomposite on a coated glass (ITO/glass) platform covered by indium tin oxide (ITO) [95], as well as 23 bases SiRNA immobilized onto the ZnO-CNT/ITO electrode by thiol link and using a physical adsorption approach. The prepared nanoplatform was used to detect *N. meningitidis* with a DPV method with a sensitivity of 20 μA and hybridization time of 45 s. In a broad range (5–180 ng/μL), the prepared DNA nanosensor demonstrated linearity.

Nanoflowers such as ZnO have also been used for magnetite detection by two separate researchers. Tak et al., using a hydrothermal method, prepared flower nanostructures such as zinc oxide (ZnO). Cyclic voltammetry (CV) and electrochemical impedance spectroscopy (EIS) have been investigated for the interaction of mechanically immobilized SiRNA probe on nanostructured ZnO [96]. The manufactured DNA nanosensor was able to measure a wide range of 5–240 ng/μL complementary target ss th-DNA with reasonable linearity of R = 0.98, high sensitivity of 168.64 μA/ng), and a low LOD of approximately 5 ng/μL.

In another work, for the specific interaction with DNA from pathogenic Leptospirosis, Perumal et al. described the fabrication of zinc oxide nanoflowers loaded by Au NPs [97]. As calculated through impedance spectroscopy, the LOD of the nanosensor achieved about 100 fM.

### 3.5. Polymeric Nanostructures

Polymeric structures have been shown to be important to ensure the success of sensors because their characteristics allow the responsiveness and efficacy of the sensing system to be adapted to different conditions and also to be increased [98].

*N. meningitidis*, *Haemophilus influenzae* type b (Hib), and *S. pneumonia*, particularly in developing nations, are the three most prevalent pathogens responsible for most bacterial meningitis [80]. A microfluidic hybrid of paper/polymer that integrated with LAMP was reported by Dou et al. for highly sensitive and specific multimodal diagnosis of these three major types of bacterial meningitis [99]. The LOD of a few DNA copies per LAMP zone for the three mentioned pathogens was achieved within just 1 h, without any advanced laboratory instruments. Moreover, these three types of artificial cerebrospinal fluid (ACSF) spiked micro-organisms were specifically observed at the same time, avoiding complicated sample preparation processes using the current approach. The hybrid microfluidic platform was shown to have a much longer lifespan in this study in comparison to the non-hybrid microfluidic paper-free platforms over a time of 3 months.

In the presence of 1-ethyl-3-(3-dimethylaminopropyl) carbodiimide hydrochloride and N-hydroxysuccinamide, Tiwari et al. coupled affinity-purified rabbit anti-glycolipid antibodies (IgG) to liposome nanoparticles (200-400 nm) to prepare a functioning tuberculosis (TB) detection reagent [100]. The assay was shown to be successful in detecting glycolipid antigens. Tuberculosis was found in clinical samples from patients with active TB with analytical sensitivity as low as 1 ng/mL, 97.4 percent clinical precision, and 96.9 percent specificity.

A rapid and effective diagnostic test for detecting *Mycobacterium tuberculosis* antigens in serum samples was stated by Tiwari et al. [101]. The polyclonal antibodies were attached to the liposomal particles in the presence of TB antigens. The liposome nanosensor developed in this study demonstrated 97.48 percent sensitivity and 95.79 percent specificity. Lipid-complexed amphotericin B (Abelcet (ABLC)) and liposomal amphotericin B (AmBisome (AmBi)) have also been shown to be effective against coccidioidal meningitis in rabbits [102].

### 3.6. Carbon Nanotubes

CNT is a good starting material for the production of super-miniaturized chemical and biological sensors due to the high response of the electrical properties of nanotubes to molecules bound on their surface and the special unit surface that allows for this great specificity [103].

Bacterial meningitis of the human brain is a life-threatening disease caused primarily by *Neisseria meningitidis*, leading to many complications, including brain injury or even death [104]. To reduce this problem, Dash et al. reported a nanoplatform of carbon/1-octadecanethiol-carboxylated MWCNTs to create a DNA nanosensor for the identification of bacterial meningitis caused by *Neisseria meningitides* [105]. The carbon composite electrode was used to covalently attach the 19-mer ssDNA probe, 5′-amine-labeled. The DNA nanosensor displayed elevated pathogenic sensitivity and can differentiate oligomer targets between complement, noncomplement, one base mismatch, and triple base mismatch. The LOD and sensor specificities were nearly 68 pM and 38.095 (μA/cm^2^)/nM, respectively.

A gene-based genosensor was described in another study from the Dash group by immobilizing a 5′-amino-modified 19-mer ssDNA probe on a composite electrode of carbon-mercaptooctadecane/carboxylated MWCNTs and hybridized with 2.5–40 ng/6 μL ssG-DNA of *N. meningitis* from Cerebrospinal fluid (CSF) in patients suspected of meningitis [106]. LOD and the sensitivity of the genosensor was 2 ng and 3.762 μA/ng of ssG-DNA for *N. meningitidis* with DPV. Interestingly, after 6 months of storage at 4 °C, the genosensor dropped to just 12% of its original DPV current.

### 3.7. Quantum Dots

The quantum dot area has now grown to include different groups of nanoparticles with various core, shell, or passivation chemistry forms. Such variations may have a profound impact on the optical properties of the resulting structures and their possible bioactivity. QDs for imaging and sensing applications such as brain disorders and infections have been used [107].

*Staphylococcal meningitis* is caused by the bacterium staphylococcus. It normally occurs as a consequence of surgery or as an inflammation that progresses through the bloodstream from another place, when it is infected by Staphylococcus epidermidis or *Staphylococcus aureus* bacteria [108]. In this respect, by using citric acid and ethylenediamine and hydrothermal method, Safardoust-Hojaghan et al. prepared graphene quantum dots (GQDs) for the detection of Staphylococcus aureus and *E. coli* [109]. Graphene quantum dots have been shown to be susceptible to *Staphylococcus aureus* and *E. coli* at low concentrations.

For the precise DNA detection of meningitis, Gupta et al. developed tungsten disulfide quantum dots (WS2 QDs) loaded in a genochip [110]. The nanosensor was used to identify target DNA and showed a broad linear answer against the target DNA in the range of 1 nM-100 μM. The LOD of the proposed nanosensor target DNA was 1 nM.

In another groundbreaking work, Wang et al. used fluorescent QDs for the simultaneous and fast identification method for *E. coli*, *S. aureus*, and *Vibrio parahaemolyticus* pathogens [111]. First, three peptides that could precisely classify the three foodborne pathogens were integrated to create an immunomagnetic nanoprobe to catch three forms of target bacteria with magnetic nanoparticles, and then three QD probes were added, which developed a sandwich structure. The residual fluorescent signal in the supernatant decreased by magnetic separation when the three quantum dot probes were directly coupled with the three pathogenic bacteria. The linear range was 10-107 cfu/mL, and the LODs of, *S*. *aureus, E. coli* and *V. parahaemolyticus* in the buffer were, 5.407, 2.460, and 3.770 cfu/mL, respectively.

## 4. Conclusions and Perspectives

The brain displays complex physiology that is impossible to be invaded with conventional drug delivery vehicles. With the advent of nanotechnology, it has become possible to permeate the BBB to deliver the drug for infections. Nanomaterials have been very promising because of their flexible properties, biocompatibility, safety, and biodegradation. The use of nanoparticles for the infectious diseases of the brain is a potential prospect in this era with novel modifications. The molecules with low hydrophobicity can also be delivered by incorporating them into suitable nanostructures. Additionally, the receptors on the surface of the bacteria can be exploited for delivering the anti-infectious agent directly, without posing serious side effects to surrounding tissues. However, with the latest developments, safety concerns must be addressed before clinical implications. In order to resolve the failure of the nervous system and brain infections, nanotechnology provides interesting ways to solve the major difficulties connected with diagnosis. For the diagnosis of infectious diseases, especially meningitis, nano-scale devices are highly promising for safe, efficient, and targeted anti-inflammatory agents and other biological compounds. Of course, certain issues such as being able to cross the BBB need to be solved by diagnostic instruments, and developments in nanotechnology will lead to improved diagnosis of brain infections. To date, much of the work in this area has been carried out in different animal models, and the next important move in pushing this area forward is to efficiently work in clinical trials, thus resolving any possible nanotoxicological concerns.

## Figures and Tables

**Table 1:** Different nanoparticles for treatment of brain infections and meningitis.

Nanostructures	Composition	Loaded Moiety	Disease	Outcome	Ref.
Liposomes	Clodronate	---	Encephalitis	Depletion of infiltrating macrophages and monocytes and reduced seizures compared to control group	[31]
Liposomes	Glycerylcaldityl-tetraether	Vancomycin	Meningitis	Increased bioavailability	[32]
Liposomes	Polypyridyl rhutenium complexes	Rabies virus glycoprotein derived peptide	Cyptococcal encephalitis	High fungicidal effect, enhanced penetration across BBB	[33]
Gold NPs	Potassium gold (III) chloride	Curcumin	Granulomatous amoebic encephalitis	Enhanced amoebicidal activity	[34]
Gold NPs	Potassium gold (III) chloride	Amphotericin B, fluconazole, nystatin	Granulomatous amoebic encephalitis	Enhanced bioactivity of drugs	[35]
Magneto-plasmonic liposomes	Iron (III) chloride hexahydrate, polyethylene-glycol (PEG)	Tenofovir disoproxil fumarate	Brain-targeted HIV treatment	Enhanced transmigration across BBB with desired therapeutic activity	[36]
Nanogel-PAMAM dendrimer	Polyethylenimine (PE) crosslinked with PEG	Nucleoside reverse transcriptase inhibitors	Brain-targeted HIV treatment	Inhibited HIV-1 in macrophages with significant accumulation in brain	[37]
Polymeric nanoparticles	Polybutyl cyanoacrylate	Amphotericin B	Meningitis	Enhanced drug delivery across BBB	[38]
Polymeric micelles	Phosphoethanolamine, PEG	Angiopep-2 and amphotericin B	Meningoencephalitis	Improved therapeutic profile of drug and accumulation in brain	[39]
Polymeric micelles	Angiopep-2, PEG, phosphoethanolamine	Amphotericin B	Fungal infection	High transcytosis across BBB	[40]
**Self-assembled NPs**	CG_3_R_6_TAT peptides	Cholesteryl chloroformate	Candida albicans meningitis	Reduction in fungal count and leukocyte concentration	[41]
**Polymeric nanoparticles**	Polybutyl cyanoacrylate	Itraconazole	Cyptococcal meningitis	Promising drug delivery vehicle across BBB	[42]
